# S146L in MYC is a context-dependent activating substitution in cancer development

**DOI:** 10.1371/journal.pone.0272771

**Published:** 2022-08-26

**Authors:** John W. Hinds, Edmond J. Feris, Owen M. Wilkins, Luke T. Deary, Xiaofeng Wang, Michael D. Cole

**Affiliations:** 1 Department of Molecular and Systems Biology, Geisel School of Medicine at Dartmouth College, Hanover, New Hampshire, Unites States of America; 2 Dartmouth Cancer Center, Dartmouth-Hitchcock Medical Center, Lebanon, New Hampshire, Unites States of America; 3 Center for Quantitative Biology (CQB), Geisel School of Medicine at Dartmouth, Lebanon, New Hampshire, Unites States of America; CNR, ITALY

## Abstract

*MYC* is one of the most dysregulated oncogenes and is thought to be fundamental to tumor formation and/or maintenance in many cancer types. This dominant pro-tumor activity makes MYC an attractive target for cancer therapy. However, MYC is a transcription factor lacking enzymatic activity, and the structure of one of its two domains is unknown e.g., its transactivation domain. Consequently, few direct MYC-targeting therapies have been developed, and none have been successful in the clinic. Nevertheless, significant effort has been devoted to understanding the mechanisms of oncogenic MYC activity with the objective of uncovering novel vulnerabilities of MYC-dependent cancers. These extensive investigations have revealed in detail how *MYC* translocation, amplification, and other upstream perturbations contribute to MYC activity in cancer. However, missense mutations of the *MYC* gene have remained relatively understudied for their potential role in MYC-mediated oncogenesis. While the function of several low-frequency mutations in MYC have been described, our understanding of other equally or more frequent mutations is incomplete. Herein, we define the function of a recurrent missense mutation in MYC resulting in the substitution S146L. This mutation enhances the interaction between MYC and its cofactor TRRAP and may enhance oncogenic MYC activity in certain cellular contexts.

**Implication:**

Our results fortify the mechanistic understanding of oncogenic MYC and may indicate a novel prognostic marker for patients whose tumors harbor the somatic mutation resulting in MYC S146L.

## Introduction

*MYC* is a proto-oncogene that potently drives tumorigenesis and is the most commonly amplified gene in human cancer [1]. The overexpression of *MYC* observed in approximately 70% of cancers is thought to be a critical step in tumorigenesis, and many of MYC’s specific oncogenic functions have been well defined [2]. Despite exhaustive research into the mechanisms of MYC-mediated oncogenesis, transcriptional control, and regulation of pro-tumor cellular functions, our understanding of MYC-induced tumor initiation and maintenance remains incomplete; however, the ability of dysregulated MYC to reprogram gene expression is undeniably linked to cellular transformation [2–5]. As such, the inhibition of MYC has been established as a powerful anti-cancer strategy *in vivo* [6–8]. Unfortunately, no clinically viable direct MYC-targeting therapies exist because the transactivation domain (TAD) of MYC is inherently disordered and lacks known therapeutically exploitable sites. Furthermore, the MYC domain with a known structure e.g., DNA binding domain (DBD), shares homology with many other transcription factors, complicating selective drug design. Accordingly, current research focuses on both directly and indirectly targeting MYC by blocking *MYC* expression and disrupting MYC interaction with various binding partners. Importantly, the precise mechanisms regulating the interaction of MYC with its cofactors are not fully defined [9]. Until this lack of understanding is addressed, MYC-targeting therapies will be limited in both feasibility and efficacy. We recently reported that the essential MYC cofactor TRRAP (Transformation/transcription domain-associated protein) forms a critical interaction within the MYC TAD, which may represent a viable strategy for targeting MYC in cancer by inhibiting MYC:TRRAP binding [9, 10]. To fully realize the potential of this strategy in targeting MYC-driven cancer, improved understanding of the MYC:TRRAP interaction is needed.

TRRAP is a central component of two histone acetyltransferase (HAT) complexes, the human Nucleosome Acetylase of H4 (hNuA4) complex and the SPT3-TAF[II]31-GCN5L acetylase (STAGA) complex. hNuA4 and STAGA complexes contain the histone acetyltransferases TIP60 and GCN5, respectively [11, 12]. Recruitment of these TRRAP-HAT complexes by MYC induces acetylation of histones within proximal nucleosomes and the subsequent relaxation of chromatin [13]. This modified chromatin then serves as a platform for other transcription factors, ultimately resulting in enhanced gene expression. Binding of MYC to these TRRAP-HAT complexes occurs at a region of homology among MYC-family proteins known as MYC-box 2 (MB2, residues 128–144), and deletion of this binding motif (ΔMB2) reduces both TRRAP binding and MYC function [9, 10, 14, 15].

To enhance our understanding of MYC:TRRAP interaction at MB2 with the eventual goal of developing a MYC:TRRAP inhibition strategy, we employed a technique devised to quantitatively determine the contribution of specific MB2 residues to MYC:TRRAP binding [10]. Additionally, we also interrogated the effect of two common cancer-associated MYC substitutions within and proximal to MB2: F138C and S146L. As such, we describe our findings that the MYC substitution S146L promotes MYC:TRRAP binding and represents a context-dependent gain-of-function mutation in cancer development.

## Materials and methods

### Cell culture

All cells were cultured in a humidified incubator at 37˚C with 5% CO_2_. Adherent cells were passaged by washing with PBS and sufficient incubation with 0.25% Trypsin-EDTA (Corning). Cultures were tested for mycoplasma every 6 months using the Lonza MycoAlertTM Detection Kit #LT07-318.

HEK293FT cells were cultured in IMDM (Caisson Labs) supplemented with 10% fetal bovine serum (FBS), 6mM L-glutamine, 0.1M non-essential amino acids (NEAA) (Corning), 500μg/mL Geneticin (Caisson Labs), 1% Pen-Strep, and prophylactic plasmocin (InvivoGen).

MCF10A cells were cultured in 50:50 DMEM/F12 (Corning) supplemented with 5% horse serum (HS) (Gibco), 6mM L-glutamine (Corning), 0.5μg/mL hydrocortisone (Sigma), 20ng/mL hEGF (PeproTech), 10μg/mL insulin (Sigma), 100ng/mL cholera toxin (Sigma), 1% penicillin/streptomycin, and prophylactic plasmocin (InvivoGen).

HCT116 and HCT116 p53^-/-^ were cultured in McCoy’s 5A medium with 10% FBS, 1% Pen-Strep, and prophylactic plasmocin.

Expi293F cell line (GibcoTM Cat# A14527, RRID:CVCL_D615) were maintained in Expi293™ Expression Medium (Gibco™) at 37°C with 8% CO2 and ≥80% relative humidity and 125 rpm shaking.

### Luminescence complementation

Luminescence complementation was performed using NanoLuc Binary Technology (NanoBiT PPI, Promega) as described previously [10]. Briefly, the coding sequence corresponding to MYC amino acids (aa) 1–190 was cloned to express a fusion with Small Binary Technology (SmBiT), while the TRRAP coding sequence for aa 2033–2283 was fused to Large Binary Technology (LgBiT). MYC 1-190-SmBiT plasmids with the MYC substitutions indicated within the text were prepared. Expi293F cells were co-transfected with adjusted ratios of each plasmid appropriate to the expression of its containing construct using ExpiFectamine™ 293 Transfection Kit per protocol (Gibco™). Cells were transfected in flasks in batches of various volumes at 3x10^6^ cells/mL. A Bluescript KS+ plasmid (Addgene) was used as carrier DNA when needed, and a pcDNA3.1 EGFP plasmid (ThermoFisher) was used as a fluorescence reporter to determine transfection efficiency. Transfected Expi293F cells were plated on 96-well white plates with clear bottoms (Greiner). White light-reflecting film (USA Scientific) was used to cover the bottom of the plates for luminescence measurements. Black light-absorbing film was used to cover the top of the plates for fluorescence measurements. All measurements were taken on a SpectraMax i3 instrument (Molecular Devices).

### Lentivirus preparation

HEK293FT cells were cultured to ~90% in 15-cm plates fed with 15 mL IMDM (Caisson Labs) + 10% FBS (HyClone), 1% penicillin/streptomycin, and 2 mM caffeine. Cells were transfected with 10 ug transfer plasmid, 6 ug psPAX2 (Addgene #12260), and 4 ug pMD2.G (Addgene #12259) using LipoD293 (SignaGen) following the manufacturer’s instructions. The following day, medium was exchanged for IMDM + 2% FBS, 1% penicillin/streptomycin, 2 mM caffeine. Eighteen hours later, viral supernatant was filtered with a 0.45-μm filter. Virus was concentrated by mixing in an appropriate volume Lenti-X concentrator (Takara) and incubating at 4˚C overnight. Virus was pelleted by centrifugation for 45 min at 1,500 x *g* at 4˚C. Viral pellets were resuspended in an appropriate volume of culture medium for the target cell line. Resuspended virus was supplemented with 1:1000 polybrene (Santa Cruz Biotechnology). When appropriate, relative virus concentration (GoStix Value [GV, arbitrary units]) was approximated using Lenti-X GoStix Plus (Takara) following the supplied instructions and associated iPhone application v2.0.7. Colon organoids were transduced with 300 GV lentivirus. HCT116 and HCT116 *TP53*^*-/-*^ cells were transduced with 1000 GV lentivirus.

### Proximity ligation assay

Proximity ligation assay (PLA) was performed with MCF10A isogenic cell lines (vector line #1, WT MYC line #1, W135E line #1, and S146L line #1). 30K cells were seeded in an 8-well chamber slide (LAB-TEK). The next day, cells were fixed in ice-cold 100% methanol and incubated at -20˚C for 15 minutes. Following fixation, methanol was removed by three 5-minute washes with PBS. Proximity ligation assay was then performed according to the manufacturer’s protocol using the kit Duolink® In Situ Red Starter Kit Mouse/Rabbit (Sigma-Aldrich). Antibodies used were V5 Tag Invitrogen #R96025, 1:500 dilution, and TRRAP AbCam #183517, 1:500 dilution. Slides were image using a Nikon fluorescence microscope with a 40X objective and a Zeiss camera coupled to ZEN lite software (Zeiss). PLA foci within cell nuclei were quantified using ImageJ, minimum 30 nuclei per field. Briefly, background-subtracted images were converted to binary images with a consistent contrast threshold. Nuclei and foci were detected using the Analyze Particles function. Only foci within cell nuclei were counted. Relative foci per nucleus was calculated as a percentage to account for any batch effect between three independent experiments.

### Immunoprecipitation

Confluent 10-cm plates of the indicated cells were placed on ice and scraped into 1 mL ice-cold PBS containing protease inhibitors (PBS+PI) (10 μM leupeptin, 10 μM Pepstatin A, 10 μM PMSF, aprotinin 1:1000 [Sigma #A6279]). Cells were collected by centrifugation and resuspended in F-buffer (10 mM Tris pH 7.05, 50 mM NaCl, 30 mM sodium pyrophosphate, 5 mM ZnCl, 10% glycerol, 1% Triton X-100) supplemented with protease inhibitors. Cells were lysed on ice for 10 minutes. Lysates were clarified at maximum speed in a microcentrifuge at 4˚C for 15 minutes. Protein concentrations were normalized to the lysate with lowest concentration by BioRad Protein Assay (BioRad # 5000006). Input samples were then collected as appropriate and prepared for western blotting by the addition of 5X sample buffer and boiling for 10 minutes. Lysates were aliquoted as needed, and antibodies or conjugated beads were added as described below. IP samples were rotated overnight at 4˚C. The following day, IP samples were washed 4X in ice-cold lysis buffer and prepared for western blotting by the addition of 5X sample buffer and boiling for 10 minutes.

### Western blotting

Samples from cultured cells were collected by first washing cells in PBS and then lysing in-well by the addition of an appropriate volume of Laemmli Lysis Buffer (LLB, 100 mM Tris pH 6.8, 25 mM DTT, 2% SDS, bromophenol blue) with protease inhibitors. Samples were then boiled for 10 minutes. Protein concentration was determined by Pierce 660 protein assay with Ionic Detergent Compatibility Reagent (IDCR, Thermo #22662 and #22663) following the manufacturer’s protocol. Lysates were normalized to protein concentration by the addition of LLB. Generally, IP samples or 20 μg of protein were run on tris-glycine-SDS PAGE minigels at 125V for 1:45 in tris-glycine-SDS buffer, or on 4–20% bis-tris gradient gels in MOPS or MES buffer 125V for 1:15 (Genscript #M00656 and #M00677). Tank blotting was performed with Immobilon-FL PVDF (Millipore # IPFL0010) in tris-glycine transfer buffer containing 20% methanol on ice at 0.35A for 2h, or at 25V at 4˚C overnight. Membranes were rinsed with water, dried, rehydrated in methanol, and blocked in 5% milk in TBS for 1hr at RT. Primary antibodies (Table 1) were diluted in 5% milk in TBST and incubated overnight on a rocker at 4˚C. Subsequently, membranes were washed 3x5 minutes in TBST. Secondary antibodies were diluted 1:10,000 in 5% milk in TBST + 0.01% SDS and incubated at RT for 1 h on a rocker. Excess secondary antibody was removed by washing 3x5 minutes in TBST and 5 minutes in TBS. Membranes were imaged and quantified on a LiCor Odyssey CLx using auto exposure, 84μm resolution, and medium quality using Image Studio software.

**Table 1:** Antibodies used for western blots.

Target	Species	Source	Cat#
*Cleaved Caspase-3 Asp175*	Rb	CST	9661
*GAPDH*	Mse	SCBT	SC47724
*IgG Rabbit Control*	Rb	CST	66362
*MYC*	Rb	CST	9402
*p21*	Rb	CST	2947
*p53*	Mse	SCBT	SC126
*Pan H4-Ac*	Rb	Millipore Sigma	06–866
*Phospho-Rpb1 CTD (Ser2) (E1Z3G)*	Rb	CST	13499
*TRRAP*	Rb	AbCam	183517
*TRRAP*	Rb	Bethyl	A301-132A
*V5 epitope*	Mse	Invitrogen	R960-25
*V5-affinity gel*	Mse	Millipore	A7345
*Vinculin*	Mse	SCBT	SC73614
*Anti-rabbit IgG DyLight 800*	Goat	CST	5151
*Anti-mouse IgG DyLight 680*	Goat	CST	5470

### Trans-well migration assay

Using a 24-well format, 1x10^6^ MCF10A cells were seeded in EGF-free and horse serum-free growth medium into uncoated permeable supports with 8.0um pores (Corning 3422). A chemotactic gradient was formed by filling the well below the support with complete growth medium. As a control, EGF and HS was omitted from the lower chamber of an additional set of samples to account for migration independent of these stimuli. 24 hours after seeding, cells that has not migrated were removed with a cotton swab. Cells that had migrated to the lower face of the permeable support membrane were stained with crystal violet (0.25% crystal violet w/v in 20% methanol) for 10 min at RT. Excess stain was removed by sufficient rinsing with water. Transwell supports were dried overnight. Membranes were imaged, and the area occupied by crystal violet-stained cells was quantified using ImageJ.

### CUT&RUN and DNA library preparation

MCF10A cells were transduced with lentiviruses expressing empty vector, WT MYC/W135E/S146L as T2A-copGFP fusions. T2A is a self-cleaving peptide that separates MYC from GFP after expression in cells. Cells were cultured for five days prior to sorting for equivalent GFP intensity (BD FACS Aria, DartLab), and subsequently cultured for three days prior to use in CUT&RUN (performed in duplicate from subsequent passages of cells). CUT&RUN was performed following the instructions for the supplied kit (Cell Signaling #86652). The following options/modifications were made to the protocol: 250,000 cells were user per reaction. Antibodies used in the reaction are listed in Table 2.

**Table 2:** Antibodies used in CUT&RUN experiment.

Target	Supplier	Cat. #	Dilution
*IgG Rabbit Control*	Cell Signaling	66362	1:20
*MYC*	Cell Signaling	9402	1:50
*TRRAP*	Bethyl	A301-132A	1:50
*Pan H4-Ac*	Millipore Sigma	06–866	1:50
*Phospho-Rpb1 CTD (Ser2) (E1Z3G)*	Cell Signaling	13499	1:50

A supplied yeast reference genome was added as recommended for the number of cells per reaction. CUT&RUN fragments were isolated by phenol/chloroform extraction. Resuspended CUT&RUN fragments were prepared for next generation sequencing using the NEBNext® Ultra™ II DNA Library Prep Kit for Illumina® and NEBNext® Multiplex Oligos for Illumina® (Dual Index Primers Set 1). Library preparation was performed using 15 cycles of amplification PCR. Size selection was performed using ProNex beads (Promega #NG2001) to collect library fragments ~150bp to ~750bp. Prepared libraries were submitted to the Dartmouth Genomics and Molecular Biology Shared Resource for next generation sequencing. Libraries were sequenced on a NextSeq500 High Output, 10 million reads/75bp paired-end reads per samples. Basecalling was performed on-instrument using RTA2. Fastq files were generated using bcl2fastq v2.20.0.422. CUT&RUN data analysis is described in S1 File.

### Human colon organoids

Organoids were derived from discarded tissues collected during tumor resection. Sample collection was reviewed and approved by Dartmouth-Hitchcock Health Institutional Review Board (STUDY02000180). De-identified samples H040 and H1110 were collected from tissue of the ascending and descending colon, respectively. Samples were collected >5 cm from the tumor site. Organoids were derived from these samples as described, with modifications [16]. Tissues specimens were washed 3X with ice-cold PBS with 50μg/mL gentamicin (Thermo). Colon epithelium separated from the submucosa was collected and minced into ~4-mm^2^ fragments. Fragments were digested in 2 mg/mL Collagenase Type I (Thermo) + 50 μg/mL gentamicin at 37˚C for 40 minutes. Colonic crypts were dissociated by sufficient pipetting. Ten mL of medium was added to neutralize collagenase. Digested crypts were collected by gentle centrifugation and subsequently digested in TrypLE for 5–10 minutes at 37˚C to isolate stem-enriched crypt termini. These fragments were then suspended in Matrigel (Corning) and plates in a 24-well plate (50 μL/well) and polymerized in an incubator for 10 minutes. Solidified matrix was overlaid with 0.5 mL expansion medium: Advanced DMEM/F12 with 10 mM HEPES, 1X Glutamax, 1X penicillin/streptomycin, 1X B27, 1X N2, 10 mM nicotinamide, 1.25 mM N-Acetylcysteine, 500 nM A83-01 (Tocris), 10 μM SB202190 (Sigma-Aldrich), 65% WRN conditioned medium (prepared from L-Cells, expressing Wnt3a R-spondin-1 Noggin, as described previously) [17], 50 ng/ml human EGF (Peprotech), 10 nM Leugastrin (Sigma), and 10 nM Prostaglandin-E2 (Peprotech). Medium was changed every 2–3 days and organoids were passaged weekly. To passage organoids, Matrigel was dissociated in ice-cold cell recovery solution (Corning) and organoids were collected by gentle centrifugation. Organoids were resuspended in TrypLE diluted 1:3 with PBS + 10 μMY-27632 (LC Labs) and incubated for 2 minutes at 37˚C, followed by pipetting 20X. Dissociated organoids were resuspended in Matrigel, split 1:3 to 1:6, and replated in 24-well plates. Replated organoids were overlaid with expansion medium + Y-27632 for two days post-passage. To prepare p53-null organoids, dissociated <10-cell clusters of organoids were prepared from confluent wells of a 24-well plate. Cell clusters from a single well were plated with the respective lentivirus in a non-treated 24-well plate in 300 uL with 10uM Y-27632 and 8 μg/mL polybrene. Organoids were spinoculated for 1 hour at 600 x *g* at 32˚C and then incubated for 2–3 hours at 37˚C before embedding in Matrigel. *TP53*-targeting guide RNA sequences were cloned into Lenti-CRISPRv2 (Addgene #82416) (guide sequences adopted from previously described p53 guide-3) [18]. Nutlin-3A was added to the medium five days following transduction to select for *TP53*^*-/-*^ organoids.

### Growth in low attachment

Growth in low attachment (GILA) was adapted from the procedure previously described [19]. Briefly, cells were seeded into 6-well or 96-well ultra-low attachment plates (Corning 3471 or 3474) at 30,000 cells in 2mL growth medium or 1,000 cells in 100μL growth medium per well, respectively. Single wells were used for assays in 6-well plates, while cells were plated in sextuplet in 96-well plates. Cells were cultured for seven days. Following the growth period, surviving cells were assayed using Cell Tier-Glo 2.0 (Promega). For all assays, Cell Tier-Glo 2.0 reagent and culture plates were equilibrated to room temperature for 30 minutes. For 96-well plates, 100μL of reagent was added to the wells and mixed thoroughly by pipetting, then incubated for 10 minutes at RT. 150μL of reaction mixture was then transferred to white, clear bottom, chimney-well 96-well plates (assay plates, Greiner Bio-One) and analyzed on a SpectraMax i3X (Molecular Devices) for luminescence (all wavelengths, 1mm read height, 500ms integration). For 6-well plate-based experiments, cells were collected in sample tubes with brief centrifugation and resuspended in 400μL of the resulting supernatant. 400μL of Cell Tier Glo 2.0 reagent was then added to the resuspended cells. To promote lysis, samples were aspirated though a 25-gauge needle with a 1mL syringe ten times. Following a ten-minute incubation, samples were diluted 1:10 with Cell Titer-Glo 2.0 reagent. 150μL of diluted reaction was then added to assay plates and analyzed as before in technical triplicate.

### Statistics

All statistical analyses were performed with GraphPad Prism using the tests indicated. Statistical analysis of genomics data was performed as described by Owen Wilkins in the Dartmouth Center for Quantitative Biology Data Analytics Core. For RNA-seq, genes expressing few than 10 counts across all samples were filtered prior to differential expression analysis. Data sets described in this study are available in the Gene Expression Omnibus under accession numbers GSE197323, GSE197327 and GSE197330.

## Results

**Fig 1:**
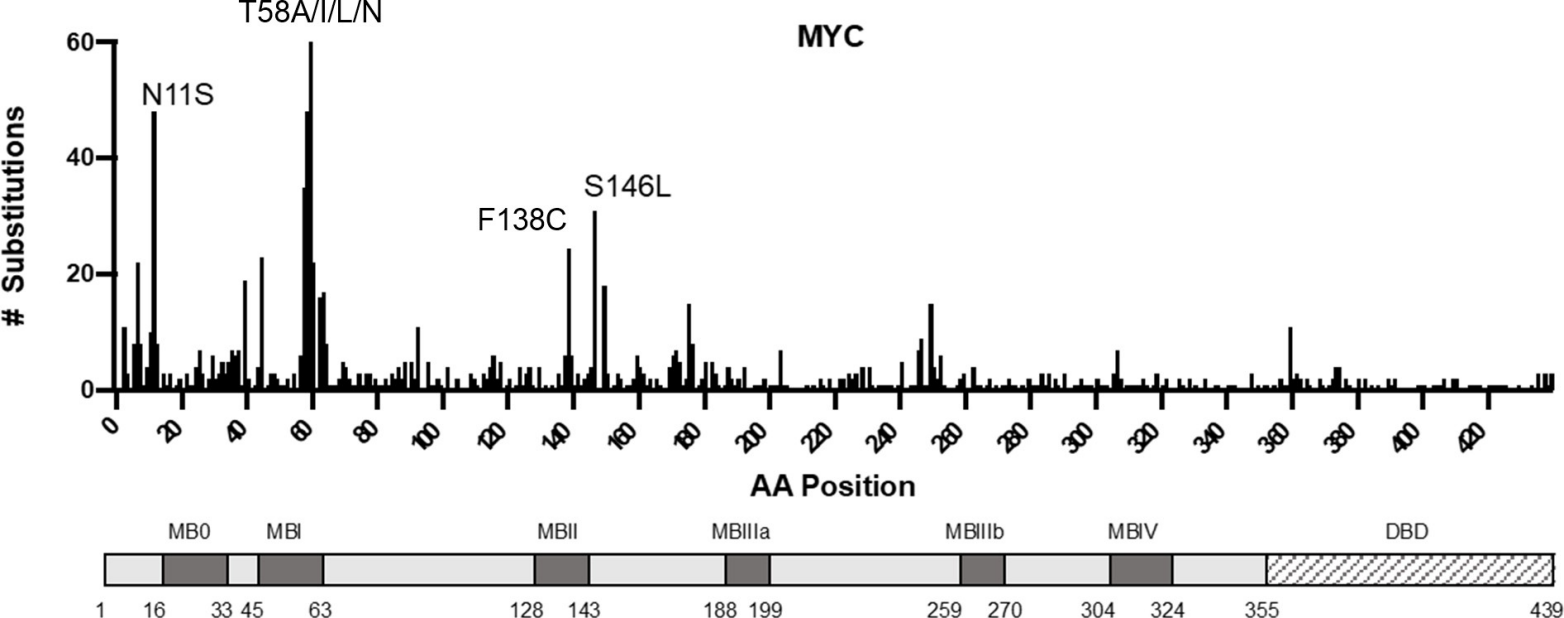
MYC substitutions observed in human cancer. The plot above denotes cancer-associated amino acid substitutions observed in human cancer and their position within conserved domains of MYC. Note that MYC N11S is the result of a germline polymorphism. Data collected from cancer.sanger.ac.uk 26 April 2021.

While somatic mutation of *MYC* occurs at relatively low frequency in cancer, several common MYC mutations have well-defined oncogenic functions. For example, nonsynonymous substitution at T58 can result in MYC protein stabilization due to the loss of phosphorylation on T58 that promotes MYC turnover [20–23]. Similarly, substitution F138C has been shown to reduce cellular response to pro-apoptotic stimuli and correlate with poor prognosis in DLBCL [24]. However, the functional consequence of many other mutations remains either poorly defined or unexplored. MYC S146L (sometimes annotated as S161L due to an alternative non-ATG start codon) is the fourth most common somatic substitution in MYC found in cancer (Fig 1). Additionally, MYC S146L is the most common somatic nonsynonymous substitution–other missense mutations result in the substitution of multiple amino acids, such as T58A, I, L, or N. Indeed, the S146L mutation is consistently the result of a single C > T transition at c.437, as mapped in relation to the ATG-start coding sequence [25]. Furthermore, the S146L substitution is present in a variety of cancer types, suggesting that it may be functional as a fundamental oncogenic mechanism irrespective of tissue of origin (Table 3). However, to date, no study has investigated the potential link between S146L and MYC-driven cancer. Intriguingly, this substitution occurs within two residues of the canonical bounds of MB2, suggesting the possibility that S146L may influence the transforming properties of MB2 (Fig 1). Additionally, this substitution is predicted to be functionally associated with disease [26]. To that end, we sought to investigate the functional impact of MYC S146L and how it may influence the process of tumorigenesis and cell survival.

**Table 3:** Frequency of S146L/S161L mutations in human cancer.

Tumor Type	Occurrence
*Haematopoietic and Lymphoid*	13
*Colon/Bowel Adenocarcinoma*	9
*Head and Neck Squamous Cell Carcinoma*	3
*Uterine/Endometriod Carcinoma*	2
*Cutaneous Melanoma*	1
*Lung Adenocarcinoma*	1
*Prostate Adenocarcinoma*	1
*Squamous Cell Carcinoma*	1
*Thymic Carcinoma*	1

**Fig 2:**
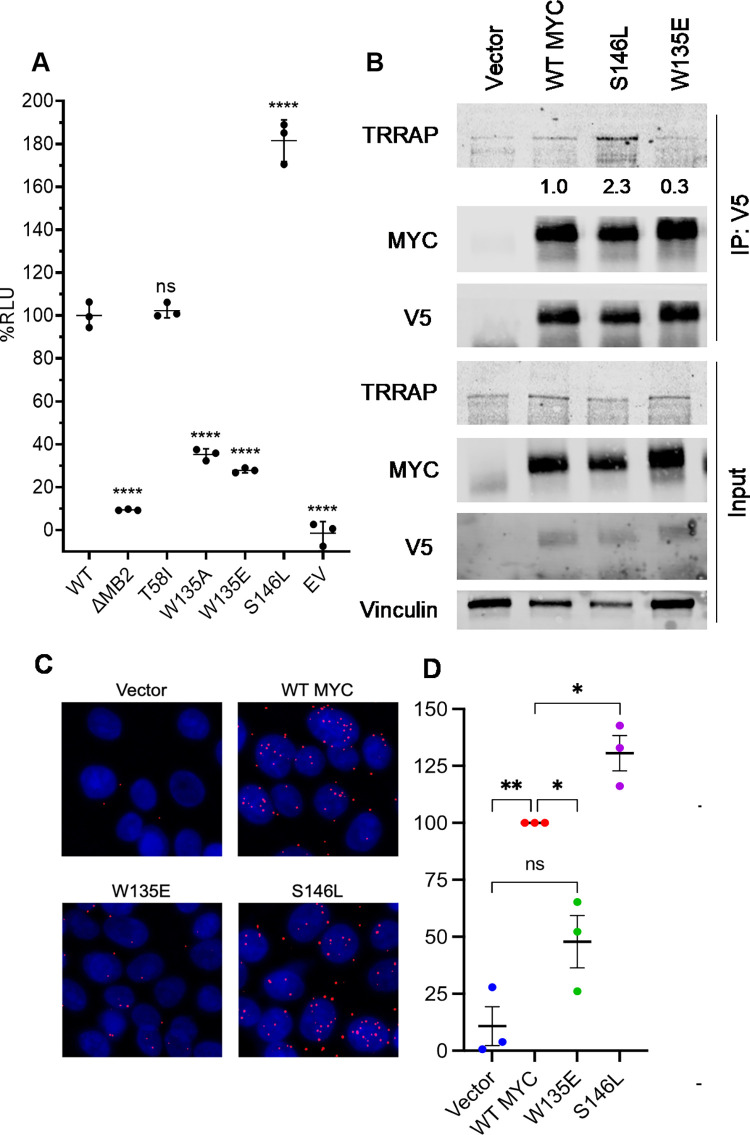
S146L enhances TRRAP binding to MYC. (A) Luminescence measurements of complementation binding assay in Expi293F cells for the indicated amino acid substitutions within MYC. The reduced-function MB2 substitution W135E served as a control for assay sensitivity. Error bars represent ±SEM (Standard Error of the Mean). (B) The S146L mutation enhances binding to TRRAP approximately two-fold. (B) Immunoprecipitation of stably expressed V5-MYC from MCF10A cells. TRRAP co-immunoprecipitation was determined by western blot. (C) Representative images of proximity ligation assay (PLA) results between V5 epitope (MYC) and TRRAP for MCF10A cells expressing the indicated MYC substitutions. (D) Quantification of results of three independent experiments (≥30 nuclei per field). PLA foci for each group were calculated as a percentage relative to WT. Quantification indicates ratio of IP TRRAP intensity to IP MYC intensity. Statistics: Holm-Šidák test. * p < 0.05, ** p < 0.01, *** p < 0.001, **** p < 0.0001.

We first determined the effect of MB2 residue substitution and S146L on MYC:TRRAP binding, postulating that due to the proximity of this residue to MB2 and its recurrence in cancer, it may induce a gain-of-function by modifying TRRAP binding to MYC. Using a split luciferase-based approach [10], we determined that the MYC S146L mutant confers an approximately two-fold greater affinity for TRRAP compared to WT (Fig 2A). Importantly, the loss-of-function W135E substitution significantly reduced MYC:TRRAP binding using this technique (Fig 2A) [10, 27, 28]. We next prepared MCF10A cells stably expressing WT MYC, MYC ΔMB2, W135E and S146L. MYC is known to autoregulate its expression in a negative feedback loop. This mechanism is best described in Burkitt’s lymphoma cells with MYC translocated to the immunoglobulin locus, where the translocated gene is highly expressed and the unaltered allele is repressed [29]. Importantly, this mechanism is likely MB2-dependent [30]. This negative autoregulatory mechanism is present in MCF10A cells, as MYC protein expressed from the endogenous MYC gene (endo) is not present when WT MYC transgene (exo) is stably overexpressed (Fig 2B). Cells expressing ΔMB2 as well as W135E MYC retain expression of endogenous MYC, suggesting that loss of TRRAP binding impairs autoregulation (Fig 2B). Notably, MYC S146L displayed endogenous MYC repression, indicating that this MYC form may preserve MB2 function sufficient to support autoregulation (Fig 2B). To further demonstrate that MYC S146L enhances TRRAP affinity compared to WT MYC, we performed proximity ligation assay (PLA) for V5-epitope tagged MYC and endogenous TRRAP in MCF10A cells. In agreement with our luminescence-based assay, more PLA foci were observed in cells expressing MYC S146L than WT, while W135E displayed reduced foci (Fig 2C and 2D). Immunoprecipitation of V5-MYC from these cells further confirmed this observation, as S146L co-purified more TRRAP protein than WT (Fig 2B). Taken together these results demonstrate that the cancer-recurrent substitution S146L promotes MYC:TRRAP binding.

**Fig 3:**
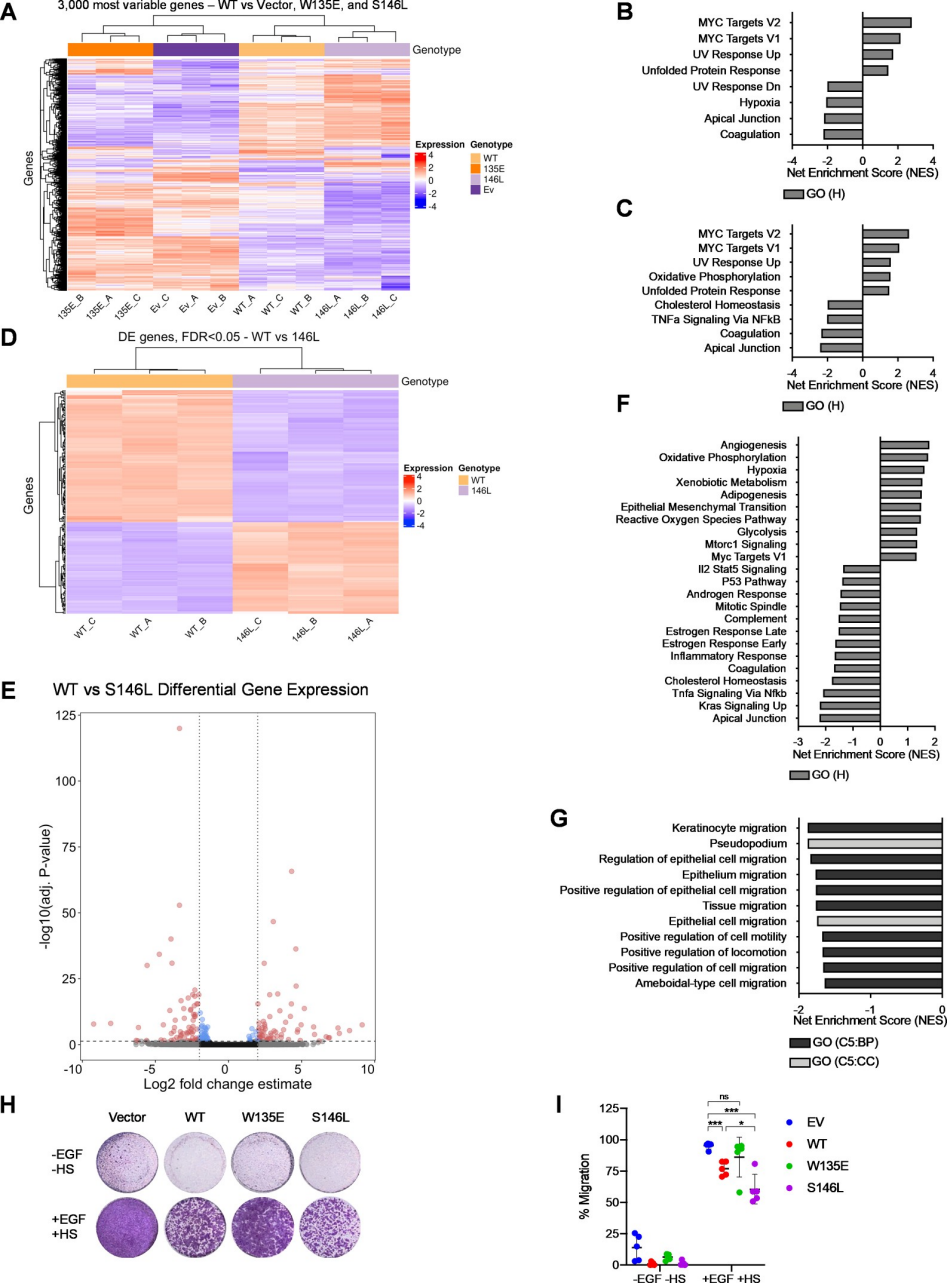
MYC S146L drives gene expression distinct from WT MYC. (A) Unsupervised hierarchical clustering of 3000 most variable genes between vector-transduced MCF10A cells (Ev), V5-tagged WT MYC, W135E, and S146L as determined by RNA-seq (n = three biological replicates). (B) Gene-set enrichment analysis (GSEA) for Hallmark gene sets differentially activated in cells expressing WT MYC compared to vector. Plot represents upregulated gene sets and four most downregulated gene sets. (P values< 0.05) except for REACTIVE OXYGEN SPECIES, MTORC1 SIGNALING, MYC TARGETS V1, and IL2 STAT5 SIGNALING (P values 0.05–0.1). (C) Gene-set enrichment analysis (GSEA) for Hallmark gene sets differentially activated in cells expressing MYC S146L compared to vector. Plot represents upregulated gene sets and four most downregulated gene sets. FDR < 0.05. (D) Unsupervised hierarchical clustering of genes with a fold change > 2 between WT MYC and MYC S146L. DE = differential expression. FDR = false discovery rate. (E) Volcano plot representing differentially expressed genes between WT MYC and MYC S146L. The y-axis in the volcano plot represents adjusted P-values that control for the FDR using the Benjamini & Hochberg method. (F) GSEA of Hallmark gene sets between WT MYC and MYC S146L. Plot represents selected most up- and downregulated gene sets. FDR < 0.1. (G) GSEA of Biological Process (BP) and Cellular Component (CC) gene sets between WT MYC and MYC S146L. Plot displays selected gene sets relating to cell motility. FDR < 0.1. (H) Representative images of a transwell migration assay of MCF10A cells expressing vector or the indicated MYC form. (I) Quantification of transwell migration as in (H) for five independent experiments. Statistics: Holm-Šidák test. ns = not significant, * *p* < 0.05, *** *p* < 0.001. EGF = epidermal growth factor. HS = horse serum.

To determine if altered TRRAP affinity results in differential regulation of gene expression, we performed RNA sequencing on MCF10A cells stably expressing WT MYC or the MYC substitutions W135E and S146L (S1 Fig in S1 File). Unsupervised clustering of the 3,000 most variable genes showed that the transcriptional profiles of vector and W135E clustered together, while more similar gene expression was found between WT MYC and S146L (Fig 3A). Gene-set enrichment analysis (GSEA) of Hallmark genes sets (MSigDB) using genes differentially expressed between vector and WT MYC and between vector and S146L confirmed that the most upregulated gene sets are MYC target genes, consistent with overexpression of the *MYC* transgene in these cells (Fig 3B and 3C). Direct comparison of gene expression between cells expressing WT MYC and MYC S146L revealed a differential gene expression profile (Fig 3D and 3E). GSEA for Hallmark gene sets between these cells revealed a multitude of differentially enriched gene sets. While no BP/CC gene sets were significant at a P<0.05, 23 gene sets demonstrated significance between P = 0.05 and P = 0.1, suggesting possible enrichment of these gene sets. (Fig 3F) Of note, S146L-expressing cells displayed a modest yet significant upregulation of MYC target genes compared to MYC WT (Fig 3F). This upregulation suggests that MYC S146L and WT MYC regulate the expression of similar genes, but the degree of transactivation stimulated by MYC S146L is greater, possibly due to enhanced TRRAP recruitment. To functionally validate these results, we tested the observation that cell migration-related genes were downregulated by WT MYC and more so by MYC S146L, consistent with prior observations that MYC may paradoxically downregulate migration when overexpressed in otherwise non-transformed cells (Fig 3G) [31]. Indeed, transwell migration of cells expressing WT MYC was significantly reduced when compared to vector, and MYC S146L expressing cells similarly displayed reduced migration compared to WT MYC (Fig 3H and 3I). These results demonstrate that WT MYC and MYC S146L may regulate certain gene sets divergently and that MYC S146L is a generally more potent transactivator of MYC target genes than WT.

**Fig 4:**
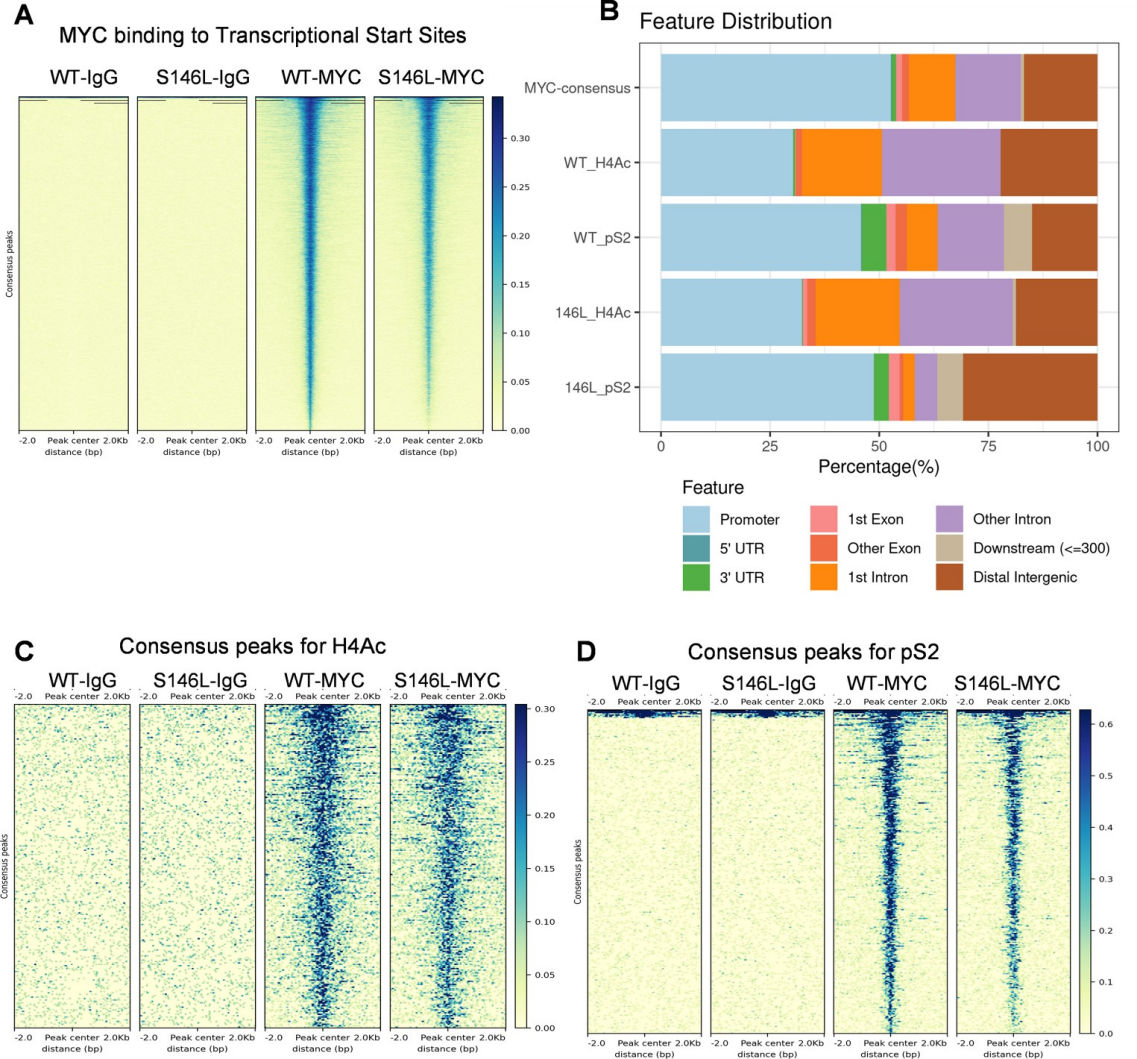
Chromatin localization between WT MYC and MYC S146L is not significantly different. (A) Heat map of reproducible MYC binding peaks observed in CUT&RUN in MCF10A cells expressing WT MYC or MYC S146L. (B) Feature distribution of consensus peaks between WT MYC and MYC S146L. (C) Differential binding analysis of MYC-overlapping H4Ac peaks observed in CUT&RUN in MCF10A cells expressing WT MYC or MYC S146L. (D) Differential binding analysis of MYC-overlapping pS2 peaks observed in CUT&RUN in MCF10A cells expressing WT MYC or MYC S146L.

Since MYC S146L putatively upregulates MYC target genes as well as other distinct gene sets compared to WT MYC, we asked if this differential transactivation was the result of altered recruitment to chromatin. To answer this question, we performed CUT&RUN (Cleavage Under Targets and Release Using Nuclease), which is functionally analogous to chromatin immunoprecipitation, followed by next generation sequencing (ChIP-seq). Differential binding analysis of WT MYC and MYC S146L showed that there was no significant difference in chromatin or transcription start site localization (Fig 4A and 4B). Furthermore, analysis of two markers of actively transcribed genes, pan-acetyl histone H4 (H4Ac) and phospho-Ser2 RNA polymerase II (pS2), demonstrated that the distribution of these marks overlapping with MYC-associated peaks was not significantly different between cells expressing WT MYC and MYC S146L at a variety of different genomic elements (Fig 4C and 4D). While a small amount of reproducible MYC-overlapping H4Ac and pS2 peaks were distinct between WT MYC and MYC S146L, binding analysis showed that these differential events did not reach statistical significance. Importantly, these data demonstrate that the differential transcriptional effects of MYC S146L at actively transcribed genes are likely the result of a mechanism independent of chromatin localization and possibly the result of enhanced TRRAP recruitment as observed in Fig 2.

**Fig 5:**
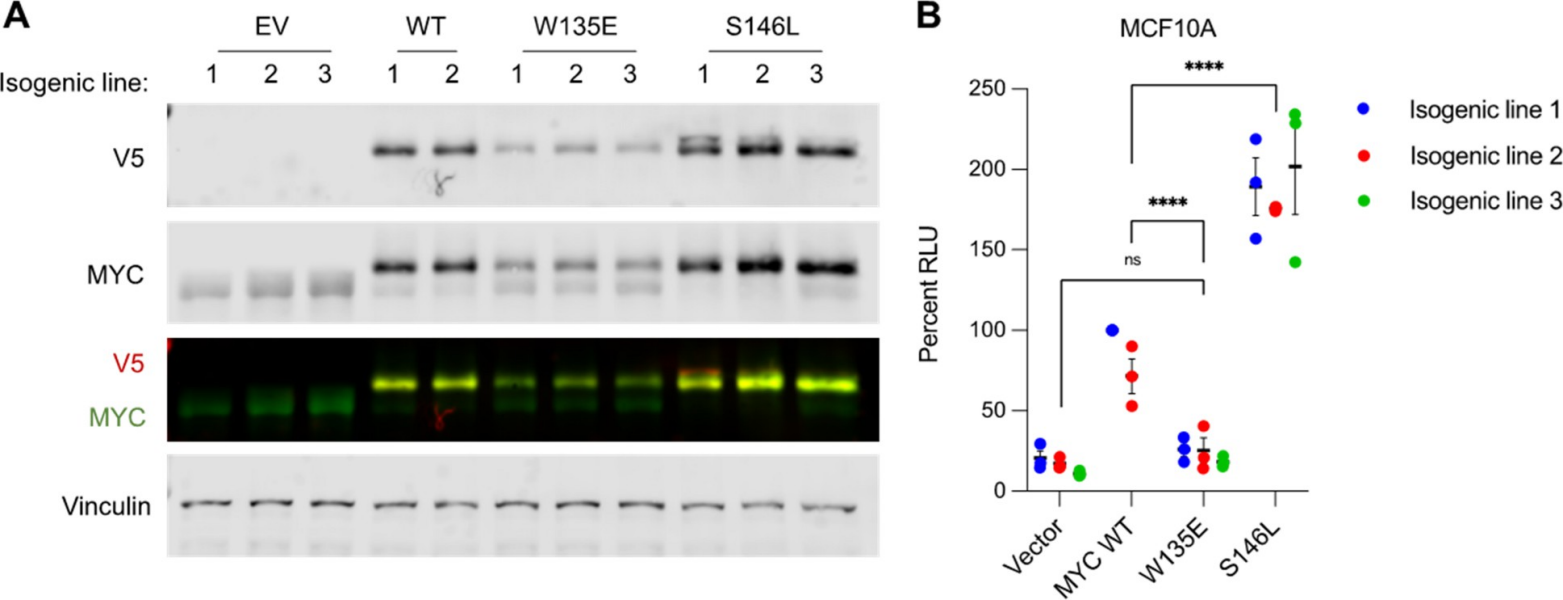
MYC S146L enhances cell survival in non-adherent conditions. (A) Western blot of V5-tagged MYC expressing MCF10A isogenic (clonally derived) cell lines. (B) Growth in low attachment assay of MCF10A cell lines represented in (A). Data represent results of three independent experiments normalized to MYC WT isogenic line 1. Percent RLU = relative luminescence units (arbitrary). Error bars represent ±SEM. Statistics: 2-way ANOVA with Tukey’s multiple comparisons test, ns = not significant, **** *p* < 0.0001.

Given that MYC S146L directs transcriptional upregulation of MYC target genes compared to WT MYC, we next tested if cells expressing MYC S146L displayed functional differences consistent with this transcriptional hyperactivation. To that end, we generated several isogenic MCF10A cell lines expressing vector, MYC WT, or the substitutions W135E and S146L (Fig 5A and S2 Fig in S1 File). To establish if cells expressing MYC S146L displayed quantifiable differences in MYC-hallmarks, we subjected these cells to growth in low attachment. Normal cells undergo senescence and/or programmed cell death upon detachment from a matrix, a process known as anoikis [32]. However, MYC expression induces anoikis resistance and non-adherent growth of MCF10A cells, both hallmarks of cancer [28]. Therefore, testing the growth of MYC-expressing MCF10A cells in low-adherence conditions is a quantitative measure of *in vitro* MYC-induced transformation. Strikingly, growth of these cell lines in ultra-low attachment conditions demonstrated that MCF10A cells expressing MYC S146L exhibited a survival advantage compared to WT MYC (Fig 5B). This observation suggests that S146L represents an oncogenic gain-of-function substitution in this already potently oncogenic protein.

Thus far, we have demonstrated that MYC S146L putatively increases MYC:TRRAP affinity, potently drives expression of MYC target genes independent of differential chromatin binding, and enhances hallmarks of MYC-driven transformation compared to WT MYC. However, much of this work has been performed in the breast epithelial cell line MCF10A. While these cells serve as an exquisite model to test the effect of oncogenic MYC, none of the known instances of MYC S146L in human cancer occur in tissues of the breast (Table 3). While the argument can be made that MYC S146L serves as a tissue-agnostic tumor promoter due to the diversity of cancer types it is observed in, we nevertheless sought to interrogate the effect of this substitution in a model more representative of human disease. To that end, we chose to establish primary human colon organoids as a model for cellular transformation induced by MYC, since MYC S146L is found in malignancies of the large intestine. These organoids are derived from the LGR5+ (leucine-rich repeat-containing G-protein coupled receptor 5) stem cell population of colonic crypts within adjacent normal tissue collected during the resection of colorectal tumors. Importantly, these organoids recapitulate the structural and functional characteristics of normal colonic tissue, making them an attractive model for the study of disease, such as the development of cancer [33]. To observe the effect of MYC expression on growth and morphology of these organoids, we transduced dissociated cells with equivalent amounts of lentivirus carrying vector, WT MYC, W135E, or S146L in frame with the self-cleaving peptide T2A fused to copepod GFP (copGFP). The T2A peptide allows the synthesis of MYC with a small C-terminal tail instead of as a MYC-GFP fusion protein. The quantity of lentivirus used for transduction resulted in the initial production of a mixed population of cells, expressing low and high expression of MYC-T2A-copGFP. This heterogenous expression allowed us to observe relative fitness of cells with different levels of transgenic MYC expression within each organoid population. GFP served as an indicator of transgenic MYC expression in this system. Organoids were imaged two days post-transduction to observe baseline GFP expression among transduced organoids (S3A Fig in S1 File). Intriguingly, at nine days post-transduction, organoids transduced with empty vector, WT MYC, and MYC W135E maintained GFP expression, while those transduced with MYC S146L lost a majority of GFP expression (S3B Fig in S1 File). This observation suggests that MYC S146L is negatively selected in these organoids since relative GFP positivity markedly declined relative to WT MYC (S3C Fig in S1 File). Because these cells are derived from primary normal tissue, we hypothesized that MYC S146L may preferentially induce oncogene-induced senescence (OIS) or apoptosis in these cells. While MYC-induced OIS is observed in some contexts, induction of apoptosis is a well-established function of MYC [34, 35]. As such, increased OIS or apoptosis upon MYC S146L expression in these organoids would be consistent with our previous observations that this substitution stimulates MYC gain-of-function. To test this hypothesis, we transduced organoids derived from the same patients with knockout of the tumor suppressor gene, *TP53*. Interestingly, the relative loss of GFP positivity in MYC S146L expressing organoids was modestly attenuated, suggesting that the negative selection observed in *TP53* wild-type cells is at least partially p53-dependent (S3D-S3F Fig in S1 File). Western blotting of these organoids 14 days post-transduction confirmed relative trends in MYC expression. However, it should be noted that these blots cannot account for heterogeneity or distribution of transgenic MYC expression levels in individual organoids (S3G Fig in S1 File). Taken together, these observations support the notion that MYC S146L induces OIS and/or apoptosis to a greater extent than WT MYC, albeit via mechanisms that are both p53 dependent and independent.

**Fig 6:**
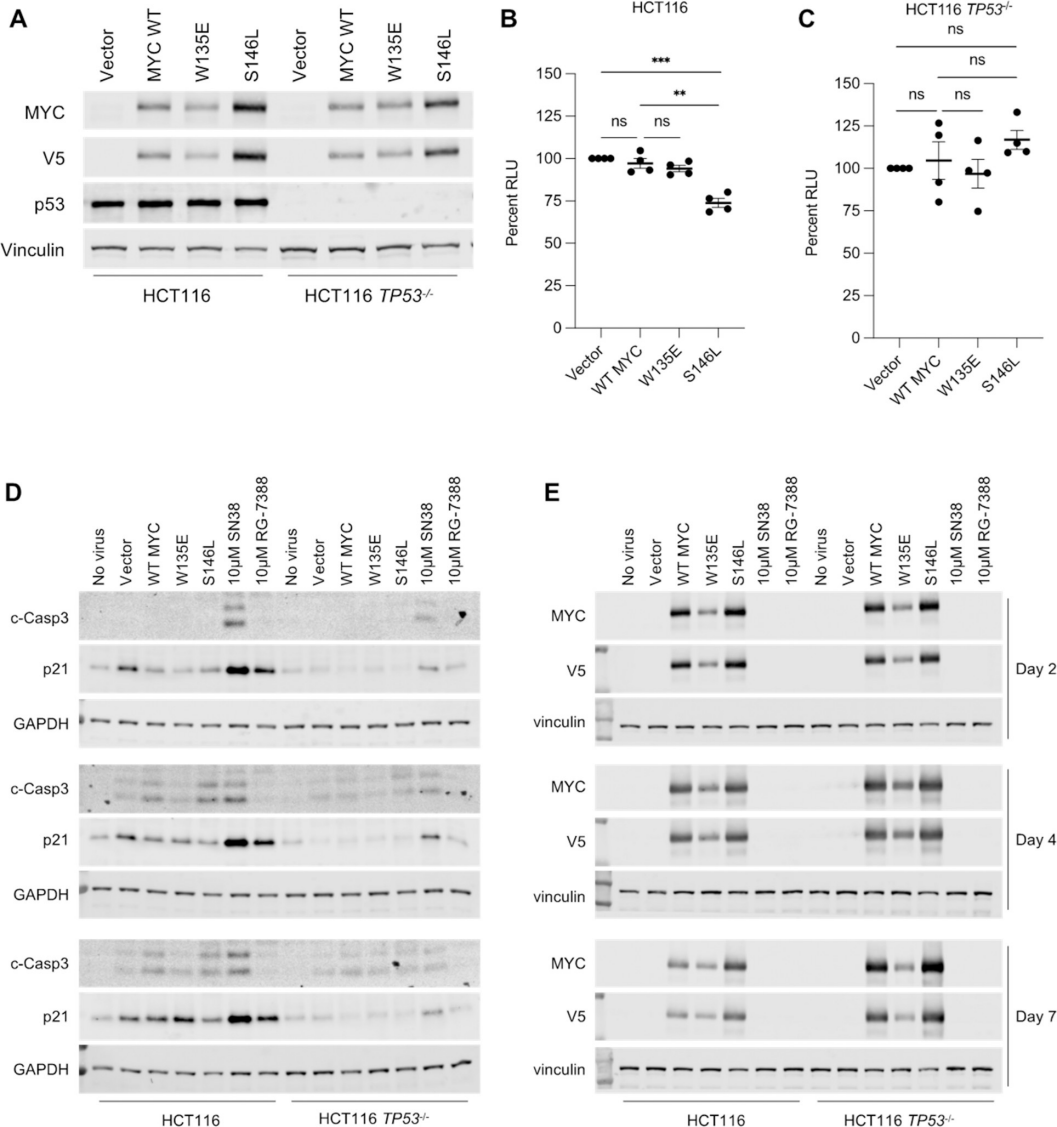
Growth inhibitory effect of S146L in HCT116 cells is p53 dependent. (A) Western blot confirming stable expression of V5-MYC WT, W135E, AND S146L in HCT116 and HCT 116 *TP53*^-/-^ cells. (B and C) growth in low attachment of the indicated cell lines stably expressing WT MYC, MYC W135E and MYC S146L relative to vector. Percent RLU = relative luminescence units (arbitrary). Statistics: Holm-Šidák test, ns = not significant, ** *p* < 0.01, *** *p* < 0.001. n = four independent experiments. (D and E) HCT116 and HCT 116 *TP53*^-/-^ cells were transduced with equivalent amounts of lentivirus expressing the indicated MYC substitutions under the control of an EF1alpha promoter. Samples were collected for analysis at the indicated timepoints and analyzed by western blotting for the indicated proteins. GAPDH and vinculin were used as loading controls. As a control for the induction of Caspase 3 cleavage (c-Casp3) and p21 expression between experimental timepoints, cells were treated for 24h with the topoisomerase-I inhibitor SN38 or the MDM2 inhibitor RG-7388 at the specified concentrations.

The results above suggest that the oncogenic effect of MYC S146L may be context dependent. Primary colon organoids undergo selection, possibly by OIS and/or apoptosis, upon MYC S146L expression, which is partially abated by the loss of the tumor suppressor p53. However, spontaneously immortalized mammary epithelial cells benefit from MYC S146L and gain distinct fitness advantages. Consequently, we deduced that MYC S146L may only be beneficial to tumor cell survival following initial cellular transformation. Subsequently, we investigated the potential for MYC S146L to enhance cancer hallmarks in an established colorectal cancer cell line, HCT116. Importantly, HCT116 cells express wild-type p53, and analysis of MYC S146L in *TP53*^-/-^ HCT116 cells should provide insight into any p53-dependent effects this substitution mutant may elicit in these cells (Fig 6A). Growth in low attachment of these cells stably expressing WT MYC and the substitutions W135E and Sl46L revealed that, surprisingly, expression of MYC S146L reduced the fitness of *TP53* wild-type HCT116 cells in non-adherent conditions compared to WT MYC, while WT MYC induced no alterations to survival (Fig 6B). Interestingly, absence of p53 abrogated this reduced fitness, as MYC S146L-expressing HCT116 *TP53*^-/-^ cells displayed no differences in survival in non-adherent conditions compared to WT MYC and a modest survival benefit over cells expressing vector (Fig 6C). However, the possibility remained that cells able to tolerate MYC S146L were selected in the process of generating stable cell lines. To test this possibility and the potential for MYC to induce OIS or apoptosis in these cells, we transduced HCT116 and HCT116 *TP53*^-/-^ cells with equivalent amounts of lentivirus carrying vector, WT MYC, MYCW135E, or MYCS146L T2A-copGFP. Samples were collected two-, four-, and seven-days post-transduction to assess the induction of the senescence regulator p21 or the apoptosis marker cleaved Caspase-3 (c-Casp3). As a positive control for the induction of senescence and apoptosis, cells were treated with the topoisomerase-I inhibitor SN38 or the MDM2 inhibitor RG-7388. While there was no distinct pattern of p21 expression among HCT116 or HCT116 *TP53*^-/-^ cells over the course of the experiment, p53 wild-type cells displayed a specific accumulation of c-Casp3 four and seven days after transduction with WT MYC or MYC S146L (Fig 6D). Importantly, p53-null cells did not exhibit significant changes in p21 or c-Casp3 accumulation between vector, WT MYC, W135E, or S146L (Fig 6D). Western blotting for MYC expression in these cells demonstrated that relative levels of WT MYC, W135E, and S146L remained consistent throughout the time course, suggesting that no differential selection based on MYC expression alone occurred (Fig 6E). Nevertheless, these results imply that the reduced fitness observed in cells expressing MYC S146L may be the result of its predisposition to preferentially induce apoptosis in an OIS-related mechanism.

## Discussion

Alterations of the MYC gene, such as translocation and amplification, are well established events that lead to MYC activation and formation of cancer. However, relatively little is known about alterations in the MYC coding sequence. While missense mutation resulting in the substitution of residues regulating MYC stability has been established as a potential mechanism promoting oncogenic MYC activity, there is a paucity of information regarding other somatic cancer-associated substitutions [20, 36]. The cancer-recurrent substitution MYC S146L occurs within three residues of the highly conserved MB2 region in the MYC TAD, which serves as a critical binding platform for TRRAP. As such, we postulated that this substitution may influence TRRAP binding to MB2. Given its presence in human disease, we further hypothesized that MYC S146L was either a passenger or gain-of-function substitution, since any deleterious effect against MYC:TRRAP binding would likely be negatively selected. To address these hypotheses, we conducted our investigation of S146L, with the ultimate finding that it may represent a context-dependent gain-of-function substitution in cancer development.

Assessment of MYC:TRRAP binding using a novel luminescence-based assay previously described [10] revealed that S146L enhanced the association of truncated forms of these two proteins (Fig 2A). Further analysis of S146L in cells showed that it enhances full-length, endogenous TRRAP binding relative to WT MYC, consistent with our initial results (Fig 2B–2D).

Increased association between MYC and TRRAP in cells likely results in a concomitant enrichment of the MYC-recruited TRRAP histone acetyltransferase complexes on chromatin. Since this mechanism is known to induce expression of target genes, we surmised that MYC S146L in turn increases gene expression when compared to WT MYC. Accordingly, gene expression analysis showed that MYC S146L not only upregulated MYC-target gene expression, but also a host of other gene sets when compared to WT (Fig 3A–3G). Interestingly, select genes repressed by MYC were further downregulated by MYC S146L. While it is unclear if TRRAP plays a direct role in MYC-mediated gene repression, MB2 is essential for such downregulation, indicating at minimum an indirect involvement of TRRAP. Our findings demonstrate that MYC S146L promotes MYC-mediated gene repression, as seen in Fig 3G–3I. Functionally, this repression results in decreased cell migration. While the reduction in cell motility upon oncogenic MYC overexpression may seem counterintuitive, overexpressed MYC in epithelial cells has been shown to downregulate specific genes that would otherwise promote cell motility. MYC overexpression in immortalized retinal pigment epithelial cell results in the downregulation of urokinase (uPA) and its receptor, uPAR, resulting in reduced migratory activity [31]. Accordingly, MCF10A cells expressing WT MYC downregulate uPA and uPAR, while MCF10A-MYC S146L cells display an even greater reduction in these genes as evidenced by RNA-seq, in agreement with the findings of the migration assay (S2 Table in S1 File). These results are therefore consistent with the notion that MYC S146L is a gain-of-function, despite the paradoxical reduction in the cancer hallmark of motility.

Given the increased association of TRRAP and enhanced expression of MYC-target genes in MYC S146L-expressing cells, we sought to determine if these two observations were directly mechanistically related. While we endeavored to demonstrate increased TRRAP occupancy at MYC S146L-associated genetic loci, attempts to analyze TRRAP-chromatin association by CUT&RUN were unsuccessful in our hands. Future work may demonstrate this interaction, potentially by using epitope-tagged TRRAP or alternative chromatin-binding analysis techniques. However, the finding that MYC S146L increases TRRAP recruitment strongly suggests that the observed MYC-induced gene expression is at least partly mediated by TRRAP-containing acetyltransferase complexes. Careful analysis of histone acetylation catalyzed by hNuA4 and STAGA using ChIP-seq or CUT&RUN may support the argument that MYC S146L specifically upregulates transcription through increased recruitment of TRRAP to chromatin. Importantly, however, we demonstrated that chromatin localization between WT MYC and MYC S146L is comparable (Fig 4A), indicating that the functional differences induced by this substitution are likely the result of altered co-factor activity rather than the result of differential localization to chromatin. Therefore, our findings are consistent with a MYC:TRRAP-regulated mechanism of transcriptional upregulation by MYC S146L.

Since MYC S146L upregulated expression of MYC-target genes relative to WT, we asked if MYC S146L also engenders cells with enhanced oncogenic traits. Assessment of MYC-induced growth in low attachment, a hallmark of cellular transformation, revealed that MYC S146L upregulates non-adherent growth compared to WT MYC (Fig 5B). Given this observation, we inferred that the S146L substitution is a gain-of-function with respect to MYC-mediated transformation. While this experiment provides robust quantitative insight into the oncogenic potential of MYC S146L, further evidence using mouse models of tumor formation may additionally support our findings and give insight in a system more recapitulative of human disease. To that end, however, we utilized human colon organoids to test for the transforming effect of MYC S146L in a system that closely models the epithelium of the human large intestine. Surprisingly, MYC S146L expression was negatively selected in these organoids, and this negative selection was only partially abated by deletion of the tumor suppressor gene *TP53* (S3A-S3F Fig in S1 File). This result suggests that MYC S146L induces oncogene-induced senescence (OIS) and/or apoptosis in these cells that is both p53-dependent and independent. While more work is needed to dissect this mechanism and the relative induction of OIS or apoptosis by MYC S146L, these results agree with the notion that cells undergo context dependent negative selection that may be partly regulated by p53.

In accordance with the finding that MYC S146L may induce context dependent OIS or apoptosis in non-transformed epithelial cells, this substitution likely evolves later in tumor evolution, rather than as a primary initiating event. The presence of S146L in established human tumors suggests that this substitution is either tolerated or beneficial at later stages of malignancy. Analysis of S146L allele frequency over time in an established tumor would more precisely indicate the bulk fitness of cells harboring this substitution compared to their WT counterparts. Additionally, many of the samples harboring S146L are derived from Burkitt’s lymphoma, within which non-translocated alleles are thought to be repressed (S1 Table in S1 File) [29]. Analysis indicating the presence of S146L in the translocated, expressed allele, rather than repressed allele would further support the notion that this substitution is not deleterious and potentially beneficial in established disease.

Due to the discrepancy between the activities of MYC S146L in MCF10A cells and colon organoids, we hypothesized that the tumor promoting function of MYC S146L is context-dependent and only beneficial following the acquisition of other oncogenic alterations. Interestingly, the established colorectal cancer cell line HCT116 exhibited reduced fitness in low attachment following expression of MYC S146L (Fig 6B). However, these cells retain functional p53. Given our observations in colon organoids, we postulated that the MYC S146L gain-of-function mechanism with respect to OIS and apoptosis may be acting in these cells as well. Analysis of growth in low attachment of p53-null HCT116 cells expressing MYC S146L showed that p53 loss abrogated the reduced fitness observed in their p53-competent counterparts, and modestly increased growth compared to cells transduced with vector (Fig 6C). Furthermore, we showed that both WT and MYC S146L expression in HCT116 cells likely induces moderate apoptosis rather than senescence as evidenced by the induction of caspase-3 cleavage (Fig 6D). These findings support the concept that MYC S146L is gain-of-function, inducing the expression of MYC-target genes including those governing pro-apoptotic mechanisms. Moreover, these observations reinforce the theory that MYC S146L likely arises later in tumor development where it may act as a tumor accelerant.

**Fig 7:**
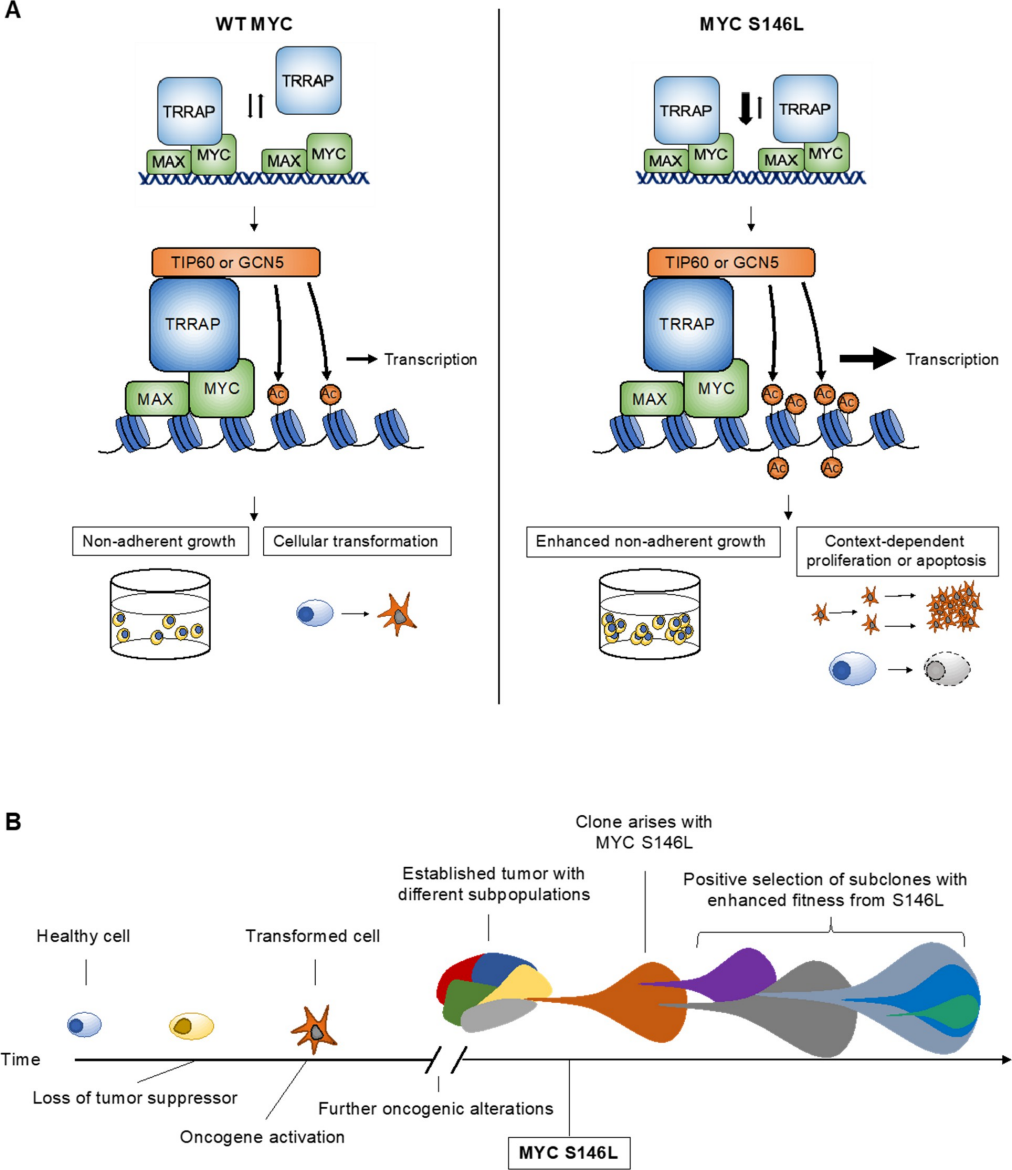
Proposed model of MYC S146L activity in transcription and cancer development. (A) Wild-type MYC likely exists in an equilibrium of TRRAP-bound and unbound states. Upon TRRAP recruitment, MYC directs the acetylation of proximal histones to promote transcription. Expression of oncogenic levels of WT MYC promotes non-adherent growth and cellular transformation. In contrast, MYC S146L promotes increased TRRAP affinity, likely leading to enhanced histone acetylation with a concomitant increase in the transcription of target genes. As a result, MYC S146L promotes increased fitness and growth in low attachment and likely augments both cell proliferation and apoptosis in a context-dependent manner. (B) Normal cells under oncogenic transformation following the deleterious genomic alterations, such as the loss of tumor suppressor function and the activation of oncogenes. Following this process, mutation of MYC S146L further activates transcription of MYC target genes. Subsequently, subclones tolerating this mutation are established and are endowed with increased fitness because of heightened MYC activity.

In considering the totality of these results, we propose the following model for the activity of MYC S146L in cancer development, as summarized in Fig 7. WT MYC recruits TRRAP, which may associate or dissociate at a given equilibrium. TRRAP-associated acetyltransferases then acetylate histone tails, relaxing chromatin, and promoting transcription. In the case of oncogenic MYC, this mechanism promotes the acquisition of cancer hallmarks, such as non-adherent growth, and ultimately leads to cellular transformation. In contrast, MYC S146L enhances TRRAP affinity and promotes recruitment of hNuA4 and STAGA complexes, which likely leads to the subsequent enrichment of acetylation and increased transcription of MYC-target genes compared to WT MYC. As a result, MYC S146L more potently drives the acquisition of cancer hallmarks than WT, while also promoting the pleiotropic effects of MYC in both cell proliferation and apoptosis (Fig 7A). The dominance of the pro-survival or pro-apoptotic effect is likely context dependent, and the MYC S146L-imbued survival benefit only arises following the establishment of malignancy and subsequent tumor evolution (Fig 7B). While more work must be done in additional disease-relevant models, our findings represent a clear indication of the potential oncogenic role of MYC S146L in cancer development.

## Supporting information

S1 File Supplemental methods. (DOCX)
