# Supplementary material for: S146L in MYC is a context-dependent activating substitution in cancer development
Source: PLoS One. 2022 Aug 26;17(8):e0272771. doi: 10.1371/journal.pone.0272771 (PMC9417018; doi:10.1371/journal.pone.0272771)
Supplement: S1 File — (DOCX) [file pone.0272771.s001.docx]

**MYC S146L is a context-dependent activating substitution in cancer development**

John W Hinds^1,2^, Edmond J Feris^1,2^, Owen M Wilkins^3^, Luke T Deary^1,2^, Xiaofeng Wang^1,2^, Michael D Cole^1,2^*

^1^ Department of Molecular and Systems Biology, Geisel School of Medicine at Dartmouth College, Hanover, New Hampshire, Unites States of America

^2^ Norris Cotton Cancer Center, Dartmouth-Hitchcock Medical Center, Lebanon, New Hampshire, Unites States of America

^3^ Center for Quantitative Biology (CQB), Geisel School of Medicine at Dartmouth, Lebanon, New Hampshire, Unites States of America

*Corresponding author: [Michael.D.Cole@dartmouth.edu](mailto:Michael.D.Cole@dartmouth.edu)

**Supplemental Methods**

**RNA isolation**

RNA was isolated from sub-confluent cultures using a Qiagen RNeasy Mini Kit (Qiagen # 74104). Briefly, buffer RLT was supplemented with the recommended concentration of DTT and added to the cell culture plate. Cells were scraped off the plate, and the lysate was disrupted with a 25-Ga needle/syringe at least five times. The subsequent procedure was performed as recommended by the manufacturer, including the optional on-column DNase digest using Qiagen RNase-free DNase (Qiagen #79256).

**RNA sequencing**

RNA was isolated from MCF10A cells expressing vector, WT MYC/W135E/S146L IRES puro as described. Quality control and sequencing was performed by the Dartmouth Genomics and Molecular Biology Shared Resource. Briefly, RNA quality was assessed on a Fragment Analyzer (advanced Analytical Technologies). Libraries were prepared from samples of high quality (RQN>9) using QuantSeq FWD methodology (Lexogen Inc., #015.96) following the manufacturer’s protocol. 250 ng of total RNA was used as input. Samples were assigned a unique index and multiplexed on a single NextSeq500 High Output flow cells (Illumina) to achieve ≥5 million, single-end 75-bp reads per sample. Base calling was performed on-instrument using RTA2. Fastq files were generated using bcl2fastq Conversion Software v2.20.0.422 (Illumina).

**RNA sequencing data analysis**

Data analysis was performed by the Dartmouth Center for Quantitative Biology Data Analytics Core. Quality of raw RNA-seq data was assessed using FastQC (v0.11.8) prior to trimming of sequencing adapters, polyA sequences, and low-quality bases from read ends using Cutadapt (v2.4). Reads were mapped to reference genome hg38 using STAR [37] (v 2.7.2b) with parameters “--outSAMattributes NH HI AS NM MD --outFilterMultimapNmax 10 --outFilterMismatchNmax 999 --outFilterMismatchNoverReadLmax 0.04 --alignIntronMin 20 --alignIntronMax 1000000 --alignMatesGapMax 1000000 --alignSJoverhangMin 8 --alignSJDBoverhangMin 1”. Quality control of aligned data was performed using CollectRNASeqMetrics (Picard Tools6). Gene-level read counts were generated HTSeq (v0.11.2) [38] with “--stranded yes” and default options. Read counts were imported into R and analyzed using R-package DESeq2 [39]. To assess relationships between samples, read counts were transformed using the regularized logarithm method implemented in DESeq2 and subjected to principal components analysis (PCA) using R function prcomp(), and unsupervised hierarchical clustering using the R-package ComplexHeatmap. The top 500 most variable genes were used for both analyses. To test for differential expression between sample groups, raw read counts were assumed to follow a negative binomial distribution, with wild-type samples used as the reference group for all comparisons. Gene-wise dispersion estimates were reviewed to confirm the selected model was an appropriate fit for the data. Option *lfcthreshold* was set to 1.0 in DESeq2 function results() to specifically test for differential expression of genes with an absolute log_2_ fold change > 1, in order to prioritize results with the largest effect sizes. Genes with a Benjamini-Hochberg adjusted P-value < 0.05 (Wald-test) were considered statistically significant. GSEA analysis was performed using the clusterProfiler R package with function gseGO() with options nPerm = 1000, minGSSize = 10. The test statistic from DESeq2 was used as the ranking metric. All differentially expressed genes that were significant at any fold change level (selected using *lfcthreshold*=0 in DESeq2 function results() ) were included in the GSEA analysis

**CUT&RUN data analysis**

Data analysis was performed by the Dartmouth Center for Quantitative Biology Data Analytics Core. Quality of raw sequence reads was assessed using FastQC (v0.11.8) and trimmed using Cutadapt (v2.4) for adapter sequences with additional parameters “--nextseq-trim 20 --max-n 0.8 --trim-n -m 1”. Reads were mapped to hg38 using Bowtie2 (v2.4.1) [40] with parameters “--local --no-mixed --no-discordant”. The resulting alignments (in BAM format) were sorted by coordinate with samtools (v1.7) [41] and filtered for unmapped or multi-mapping reads using sambamba (v0.6.8) [42]. Duplicate reads were identified and removed using MarkDuplicates (Picard Tools) [43]. deepTools (v 3.4.3) command “bamPEFragmentSize” was used to confirm expected fragment length distributions of individual samples. For MYC/MYC-V5, peaks were called using the MACS2 (v2.2.7.1) callpeak command in narrowpeak mode, using IgG IP samples as controls with parameters “-f BAMPE --keep-dup all -g 2913022398 -q 0.05”. For H4Ac and pS2, peaks were identified using MACS2 callpeak command in broadpeak mode again using IgG IP samples as controls and with identical parameters as above, as well as additional parameters “--broad --broad-cutoff 0.05”. Fraction of reads in peaks (FRiP) was calculated for each sample to assess individual quality. Called peaks were filtered against the ENCODE human blacklist (dataset: ENCFF356LFX) and further restricted to peaks demonstrating a 2-fold or greater signal increase relative to control (IgG) samples. BEDTools (v 2.29.2) command “intersect” with parameters “-a” was used to compare replicates and identify a set of reproducible overlapping peaks for each sample group. For visualization of CUT&RUN signal across specific genomic regions, BAM files from biological replicates were merged MergeSamFiles (Picard Tools) and supplied to the deepTools (v3.4.3) bamCoverage command to generate counts per million (CPM)-normalized signal-tracks (in BigWig format) with parameters “--binSize 20 --normalizeUsing CPM --effectiveGenomeSize 2913022398 --extendReads --smoothLength 60 --centerReads”. Heatmaps of normalized CUT&RUN signal in specified regions were generated using deepTools commands computeMatrix and plotHeatmap. Peak sets were annotated using the annotatePeak() function from the ChIPseeker [44] package, using a range of +/- 1kb to define promoter regions. R-package TxDb.Hsapiens.UCSC.hg38.knownGene was used to define genomic features. Peaks greater than 10kb from their closest gene were annotated as ‘”intergenic” peaks and not annotated to a gene. To facilitate differential binding analysis of individual factors, reads occurring in called peaks were counted from BAM files using the featureCounts() function (Rsubread package) with options “isPairedEnd=TRUE, countMultiMappingReads=FALSE”. Read counts were used to perform differential binding analysis of WT vs S146L samples for MYC, H4Ac, and pS2 with DESeq2. Peaks achieving a Benjamini-Hochberg adjusted P-value < 0.05 (Wald-test) were considered statistically significant. For differential binding analysis of H4Ac and pS2, peak sets were restricted to those overlapping regions also containing a MYC peak.

**S1 Fig. MYC expression in MCF10A MYC-T2A-copGFP cells.** Western blot of copGFP-sorted MCF10A cells stably expressing vector, MYC WT, MYC W135E, or MYC S146L used in RNA-sequencing analysis.

**S2 Fig. Adherent growth of MCF10A isogenic lines is consistent.** MCF10A cells were plated in 96-well plates on Day 0 and assessed for DNA content using Hoechst 33342 as a measure of cell growth on the indicated days. Dotted lines indicate the average growth fold-change of vector-transduced cells at Day 5.

**S3 Fig. Expression of MYC S146L is negatively selected in primary human colon organoids.** (A) Images of colon organoids derived from patient 1110 two days after transduction with lentivirus carrying the indicated MYC transgene. (B) Images as in (A) nine days following transduction. (C) Quantification of relative GFP positivity between days two and nine following transduction. The fold change (FC) of the ratio of GFP+ area to organoid-occupied area between days two and nine was calculated relative to WT MYC. (D-E) Data as in (A-C) for *TP53*-null organoids. (G) Western blot of organoids 14 days post-transduction indicating relative MYC transgene expression. Exo = transgenic MYC protein, endo = MYC protein produced by endogenous alleles.

**S1 Table. TCGA samples with MYCS146L mutations.** Data collected from COSMIC: cancer.sanger.ac.uk 26 April 2021, Tate *et al.* 2018.

| Sample ID | Primary Tissue | Cancer Type |
| --- | --- | --- |
| TCGA-DS-A1OB-01 | cervix | squamous cell carcinoma |
| P-0004793-T01-IM5 | endometrium | mixed serous and endometrioid carcinoma |
| 1052-01-5TD | hematopoietic and lymphoid tissue | chronic lymphocytic leukaemia-small lymphocytic lymphoma |
| 1059 | hematopoietic and lymphoid tissue | Burkitt lymphoma |
| 15_1 | hematopoietic and lymphoid tissue | B cell prolymphocytic leukemia |
| 2972 | hematopoietic and lymphoid tissue | Burkitt lymphoma |
| 2974 | hematopoietic and lymphoid tissue | Burkitt lymphoma |
| 4947 | hematopoietic and lymphoid tissue | Burkitt lymphoma |
| 6107 | hematopoietic and lymphoid tissue | Burkitt lymphoma |
| 6116 | hematopoietic and lymphoid tissue | Burkitt lymphoma |
| 6197 | hematopoietic and lymphoid tissue | Burkitt lymphoma |
| BPLL_33 | hematopoietic and lymphoid tissue | B cell prolymphocytic leukemia |
| L06 | hematopoietic and lymphoid tissue | post transplant lymphoproliferative disorder |
| QC2-19-T2 | hematopoietic and lymphoid tissue | diffuse large B cell lymphoma |
| P-0006587-T01-IM5 | large intestine | adenocarcinoma |
| T2641 | large intestine | adenocarcinoma |
| T3048 | large intestine | adenocarcinoma |
| T3048 | large intestine | adenocarcinoma |
| T3246 | large intestine | adenocarcinoma |
| TCGA-A6-5659-01 | large intestine | adenocarcinoma |
| TCGA-SS-A7HO-01 | large intestine | adenocarcinoma |
| TCGA-69-8253-01 | lung | adenocarcinoma |
| P-0006380-T01-IM5 | ovary | endometrioid carcinoma |
| 16 | pancreas | NS |
| AA2463 | peritoneum | epithelial |
| P-0012129-T01-IM5 | skin | squamous cell carcinoma |
| TCGA-EE-A182-06 | skin | cutaneous melanoma |
| P-0008184-T01-IM5 | small intestine | adenocarcinoma |
| TCGA-CV-A45V-01 | upper aerodigestive tract | squamous cell carcinoma |
| TCGA-D6-6825-01 | upper aerodigestive tract | squamous cell carcinoma |
| TCGA-H7-8501-01 | upper aerodigestive tract | squamous cell carcinoma |

**S2 Table. Downregulation of uPA and uPAR in MCF10A-MYC and MCF10A-MYC S146L cells.** uPA, urokinase. uPAR: urokinase receptor

| WT vs S146L | |
| --- | --- |
| Gene | Fold change |
| uPA | -1.67 |
| uPAR | -1.06 |
